# Virological and Biochemical Effects of Tenofovir Alafenamide in Different Patient Groups With Chronic Hepatitis B Virus Infection in Real-World Cohort

**DOI:** 10.1155/ijh/9632839

**Published:** 2025-04-15

**Authors:** Erdem Bektas, Aysenur Yilmaz, Cevat Ilteris Kikili, Kanan Nuriyev, Zulal Istemihan, Ibrahim Volkan Senkal, Ziya Imanov, Bilger Cavus, Asli Cifcibasi Ormeci, Filiz Akyuz, Kadir Demir, Selman Fatih Besisik, Sabahattin Kaymakoglu

**Affiliations:** ^1^Department of Internal Medicine, Istanbul Faculty of Medicine, Istanbul University, Istanbul, Turkey; ^2^Division of Gastroenterology, Department of Internal Medicine, Istanbul Faculty of Medicine, Istanbul University, Istanbul, Turkey

## Abstract

Hepatitis B virus (HBV) infection is an important health concern worldwide. HBV infection can lead to acute hepatitis, cirrhosis, hepatocellular carcinoma, liver failure, and death. Nucleos(t)ide analogs (NAs) form the core of the HBV treatment. The safety and efficacy of NAs in long-term follow-up are still critical issues. We enrolled 225 consecutive patients with at least 12 months of longitudinal follow-up using tenofovir alafenamide (TAF), including 39 antiviral naïve and 186 antiviral experienced patients. In the treatment-experienced group, the main reasons for switching from other NAs to TAF were renal dysfunction and osteoporosis. Renal outcome, lipid profile, virological response, and ALT normalization under the TAF treatment were evaluated. Age > 60 years, liver transplant recipients, and patients with decompensated cirrhosis were evaluated separately, as well as the total cohort. Phosphorus levels increased especially in hypophosphatemic individuals, eGFR levels also increased slightly but statistically significantly, and the remarkable improvement in eGFR stages was observed in the eGFR < 60 mL/min/1.73 m^2^ group. A minimal increase in LDL-c levels occurred after TAF treatment, which did not reach statistical significance. Total cholesterol and HDL-c levels increased significantly, while triglyceride levels remained unchanged. In the total cohort, HBV-DNA was strongly suppressed in either treatment-naïve or experienced patients. ALT and AST levels decreased with the TAF treatment, but ALT normalization rate did not change significantly. No serious adverse events associated with TAF occurred, and discontinuation was not required in the total cohort. Our findings support that TAF treatment is well-tolerated and effective in patients with chronic HBV infection.

## 1. Introduction

Hepatitis B virus (HBV) infection is one of the major global health problems that could impact millions of people, particularly in underdeveloped areas [1]. More than 275 million people are chronic carriers of the virus, even though it is a vaccine-preventable disease [2]. HBV can lead to acute hepatitis and acute liver failure, as well as progressive liver fibrosis that can cause lethal complications which are decompensated cirrhosis (DC) and hepatocellular carcinoma (HCC) without treatment [3]. Although the prevalence of chronic HBV infection and HBV-related mortality is declining, HBV infection remains a critical health problem worldwide [1].

Chronic or occult HBV infection may require specific antiviral treatment in certain conditions to prevent long-term complications and HBV reactivation. The main goal of antiviral treatment is preventing disease progression to improve survival; also, additional goals are preventing transmission from mother to child, HBV reactivation, and treatment of extrahepatic manifestations. In the treatment of HBV infection, virological, serological, biochemical, and histological responses were defined as undetectable HBV-DNA titers, loss or seroconversion of Hepatitis B antigens (HBeAg and/or HBsAg), normalization of serum transaminase levels, and decrease in necroinflammation and fibrosis of the liver, respectively [4, 5]. Interferon-based therapy and potent nucleos(t)ide analogs (NAs) such as tenofovir disoproxil fumarate (TDF), entecavir (ETV), and tenofovir alafenamide (TAF) form the basis of HBV treatment [6].

The viral suppressive effect and safety profile of antiviral agents are important issues in HBV treatment. Prior studies reported that TAF has fewer safety concerns in terms of renal function and bone health, while the antiviral effect of TAF was found to be noninferior compare to TDF [7, 8]. The guideline for the clinical management of CHB recommends TAF or ETV over the TDF treatment in patients aged > 60 years, patients with bone disease (such as osteoporosis and fragility fractures), and renal dysfunction (such as low glomerular filtration rate, hypophosphatemia, and hemodialysis) [5]. In the literature, in contrast to TAF or TDF treatment, drug resistance, albeit very low, has been reported in long-term ETV treatment [9]. Recently, some conflicting data have been provided regarding that TAF may cause dyslipidemia [10].

In this study, we aim to report the efficacy and safety data of the TAF treatment in antiviral naïve patients and patients using TAF as sequential therapy with the real-world cohort.

## 2. Method

### 2.1. Patients

This is a retrospective cohort study from a single center. A total of 225 consecutive patients above the age of 18 who were longitudinally followed for at least 12 months under the TAF treatment (once-daily 25 mg) between 2019 and 2022 were enrolled in the study from our tertiary referral gastroenterology clinic. Patients with HBeAg ± chronic Hepatitis B (CHB, HBsAg positivity persisting for ≥ 6 months, liver disease severe enough to require treatment, and replicative HBV infection), patients receiving antiviral therapy to prevent HBV reactivation while using concomitant chemotherapeutic or immunosuppressant agents (as prophylaxis, in HBsAg positive or anti-HBc positive patients), and liver transplant recipients were involved in this study. The patients provided informed consent. This study complied with the Declaration of Helsinki and was approved by the Istanbul University Istanbul Faculty of Medicine Ethics Committee (Date-Number: 2023-1697544).

### 2.2. Data Collection

The demographic, clinical, and laboratory data of the patients were obtained from their medical records. Baseline data of patients at the starting TAF treatment (Pre-TAF) and at 12 months after the TAF treatment (TAF [12 m]) were documented. Follow-up period; age; sex; the rate of patients with cirrhosis due to CHB (diagnosed by clinical, laboratory, and imaging findings); the rate of the liver transplant recipients; viral markers such as HBsAg, HBeAg, and HBV-DNA titers; alanine aminotransferase (ALT) levels; bilirubin levels; platelet counts; and albumin levels were determined as baseline patient's characteristics at the starting TAF treatment. FIB-4 scores were calculated to estimate the stage of fibrosis in all patients (< 1.45, 1.45–3.25, and > 3.25 as Ishak score Stages 0–1, 2–3, and 4–6, respectively), whereas the MELD-Na scores, Child–Pugh scores, and the rate of decompensation were noted in cirrhotic individuals [11–13]. In antiviral experienced patients, the antiviral agent that was used before TAF and the duration of use were assessed. In addition, certain causes based on EASL guideline for starting or switching to the TAF were noted [5].

**Figure 1:**
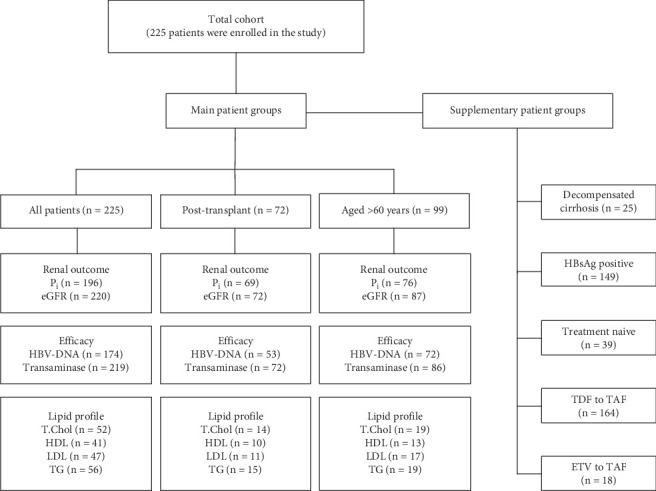
Flow chart of the patient groups (eGFR, estimated glomerular filtration rate; ETV, entecavir; HDL, high-density lipoprotein; LDL, low-density lipoprotein; *n*, number of evaluable patients; Pi, inorganic phosphorus; T. Chol, total cholesterol; TAF, tenofovir alafenamide; TDF, tenofovir disoproxil fumarate; TG, triglyceride).

Biochemical data including inorganic phosphorus (Pi) levels, estimated glomerular filtration rates (eGFR was calculated using the CKD-EPI formula), eGFR stage changes, lipid profiles (total cholesterol [TC], high-density lipoprotein [HDL-c], low-density lipoprotein [LDL-c], and triglyceride [TG] levels), and ALT levels were recorded. Pi levels (mg/dL) were classified: < 2.5 as hypophosphatemia and ≥ 2.5 as normophosphatemia. The eGFR threshold level was determined as 60 mL/min/1.73 m^2^, and the patients were divided into two subgroups, those with eGFR < 60 and those with eGFR ≥ 60. Stages of eGFR were categorized as G1–G5 based on the eGFR levels (mL/min/1.73 m^2^) which are above 90 as G1, 60–89 as G2, 45–59 as G3a, 30–44 as G3b, 15–29 as G4, and < 15 as G5. The upper limit of the normal range (ULN) for ALT (IU/mL) was defined as > 35 for men and > 25 for women based on the AASLD guidelines [4]. ALT levels were categorized as below or above the ULN for ALT, and normalization was defined as a decrease below the ULN for ALT. The virological response to CHB was evaluated by considering HBV-DNA levels (QIAGEN, *artus* HBV QS-RGQ Kit). HBV-DNA levels (IU/mL) were classified as < 31.6 and ≥ 31.6. Liver function tests, electrolytes, complete blood counts, and coagulation tests were evaluated at each visit. Complications related to chronic liver disease (decompensation, HCC, etc.) and TAF-related side effects were noted during treatment. In addition, patients with DC, patients switched from TDF to TAF and ETV to TAF, HBsAg-positive patients, and treatment-naïve patients were evaluated as separate groups. The number of evaluable patients in the main and supplementary subgroups is shown in the flow chart (Figure 1).

### 2.3. Statistical Analysis

Quantitative variables were described as the mean ± standard deviation (SD) or median (interquartile range [IQR]) based on the distribution of the data, while the categorical variables are presented as numbers and percentages. Histogram, skewness and kurtosis coefficients, the Kolmogorov–Smirnov test or Shapiro–Wilk test as appropriate, and Q-Q plots were used to determine the normality of the data. Chi-square and Fisher's exact tests were used for nominal categorical comparisons. Comparisons of dependent ordinal categorical variables were performed using the Wilcoxon signed-rank test. In addition, dependent quantitative variables were compared by using the Wilcoxon signed-rank test regarding the nonparametric nature of these variables. Missing data were not included in the statistical analysis. Statistical analysis was performed by using SPSS V26.0 (IBM Corp., Armonk, New York), and a two-tailed *p*-value of < 0.05 was accepted as statistically significant.

## 3. Results

### 3.1. Patients Characteristics

This study was conducted with a total of 225 patients, of whom 158 (70.2%) were male. The median age was 57 (48–65) years, and the follow-up period was 22 (12–30) months. At the start of the TAF, 43 (22.2%) patients were cirrhotic. Among the patients with cirrhosis, 25 patients had at least one decompensation finding. In cirrhotic patients, the MELD-Na score was 11 (9–15.5), while 18 (41.9%) were Child A, 20 (46.5%) were Child B, and 5 (11.6%) were Child C. The FIB-4 score was < 1.45 in 110 (49.7%), 1.45–3.25 in 75 (33.9%), and > 3.25 in 36 (16.2%) patients. Seventy-two of all patients were liver transplant recipients. HBsAg was positive in 149 (66.2%) and HBeAg in 10 (8.2%) patients. HBV-DNA titers were ≥ 31.6 IU/mL in 27 (15.5%) patients in the total cohort, while 12 (54.5%) in antiviral naïve patients. Forty-six (20.5%) patients in all patients had ALT levels above ULN before the TAF treatment, while 13 (35.1%) in antiviral naïve patients. The total bilirubin levels were 0.6 (0.37–0.97) mg/dL, the platelet counts were 138 × 10^3^/*μ*L (180–239 × 10^3^/*μ*L), and the albumin levels were 4.6 (4.27–4.8) g/dL.

The antiviral naïve group consisted of 39 (17.3%) patients, and 24 (61.5%) of them used TAF for prophylaxis. The median duration of antiviral usage prior to the TAF treatment was 127 (81–177) months. Patients who switched from TDF to TAF were 164 (88.2%), from ETV to TAF were 18 (9.7%), and from other antivirals to TAF were 4 (2%). Certain causes of starting or switching to TAF were hypophosphatemia (110 [48%]), eGFR < 60 mL/min/1.73 m^2^ (62 [27.6%]), osteoporosis (22 [9.8%]), proteinuria (5 [2.2%]), and others (17 [7.6%]), while 24 patients had multiple causes. The switching causes from NAs to TAF are shown in Table S1. Forty-one (18.2%) patients used TAF for the prevention of HBV reactivation. Baseline demographic, clinical, and treatment characteristics of the total cohort are presented in Table 1.

### 3.2. Phosphorus and Renal Outcome

In the total cohort, the Pi levels increased significantly from 2.48 (2.16–3.15) to 2.89 (2.5–3.32) mg/dL in the all patients group (*n* = 196) and from 2.16 (2–2.4) to 2.61 (2.36–3) mg/dL in the hypophosphatemic group (*n* = 99) (both *p* < 0.001). In the normophosphatemic group (*n* = 97), Pi levels were similar from baseline (3.2 [2.77–3.58] mg/dL) to after TAF treatment (3.18 ± 0.64 mg/dL) at the 12-month follow-up period (*p* = 0.994).

In the posttransplant (Tx) group, Pi levels followed the same trend as in the total cohort, with a significant increase from 2.4 (2.1–2.9) to 2.8 (2.55–3.16) mg/dL in the all patients group (*n* = 69) (*p* < 0.001). While Pi levels in the hypophosphatemic group (n = 43) increased from 2.17 (2.02–2.4) to 2.7 (2.48–2.9) mg/dL (*p* < 0.001), the difference in Pi levels between Pre-TAF (3.00 [2.8–3.23] mg/dL) and TAF (12 m) (3.01 [2.8–3.45] mg/dL) was also not statistically significant in the normophosphatemic group (*n* = 26) (*p* = 0.508).

The change in Pi levels from Pre-TAF to TAF (12 m) in the > 60 age group showed a similar trend to the total cohort and the post-Tx group. There was a significant increase in Pi levels from 2.55 (2.24–3.27) to 2.92 ± 0.73 mg/dL in the all patients group (*n* = 76) (*p* = 0.021) and from 2.21 (2.05–2.4) to 2.6 (2.24–2.91) mg/dL in the hypophosphatemic group (*n* = 35) (*p* = 0.002). In the normophosphatemic group (*n* = 41), the marked difference in Pi levels from Pre-TAF to TAF (12 m) was not observed (3.2 [2.7–3.61] vs. 3.25 [2.7–3.64] mg/dL; *p* = 0.936). Among hypophosphatemic patients, Pi levels normalized (> 2.5 mg/dL) in 63 (64%) of the total cohort, 32 (74%) of the post-Tx group, and 21 (60%) of the > 60 age group.

The change in eGFR levels from Pre-TAF to TAF (12 m) and eGFR stages was analyzed to evaluate the renal outcome. Among the total cohort, eGFR levels did not increase remarkably from 77.5 (55–95) to 79 (58–94) mL/min/1.73 m^2^ in the all patients group (*n* = 220), and the significant difference was not seen from 88.5 (75–99.5) to 87 (76.5–101) mL/min/1.73 m^2^ in the eGFR ≥ 60 group (*n* = 152) (*p* = 0.105 and *p* = 0.460, respectively). In the eGFR < 60 group (*n* = 68), there was a statistically significant difference in eGFR levels between Pre-TAF and TAF (12 m) (50 [41.5–53.5] and 54 [44.25–60] mL/min/1.73 m^2^, respectively; *p* < 0.001). The proportion of eGFR stages in all patients group was not statistically different from baseline values (*p* = 0.536). The difference in eGFR stages between Pre-TAF and TAF (12 m) in the < 60 eGFR group was a statistically significant improvement (*p* < 0.001).

The change in eGFR levels from Pre-TAF to TAF (12 m) in the post-Tx group was not statistically different in the all patients group (*n* = 72) and the ≥ 60 eGFR group (*n* = 43) (66 [51–88.5] vs. 71 [57–85.75] and 82 [68–99] vs. 81 [74–97] mL/min/1.73 m^2^; *p* = 0.155 and *p* = 0.822, respectively), whereas there was a statistically significant difference in the < 60 eGFR group (*n* = 29) (50 [43–52] vs. 55 [45–60] mL/min/1.73 m^2^; *p* = 0.016). There was no statistical difference between Pre-TAF and TAF (12 m) in the post-Tx group (*p* = 0.300), whereas an improvement in eGFR stages was found in the < 60 eGFR group (*p* = 0.016).

**Figure 2:**
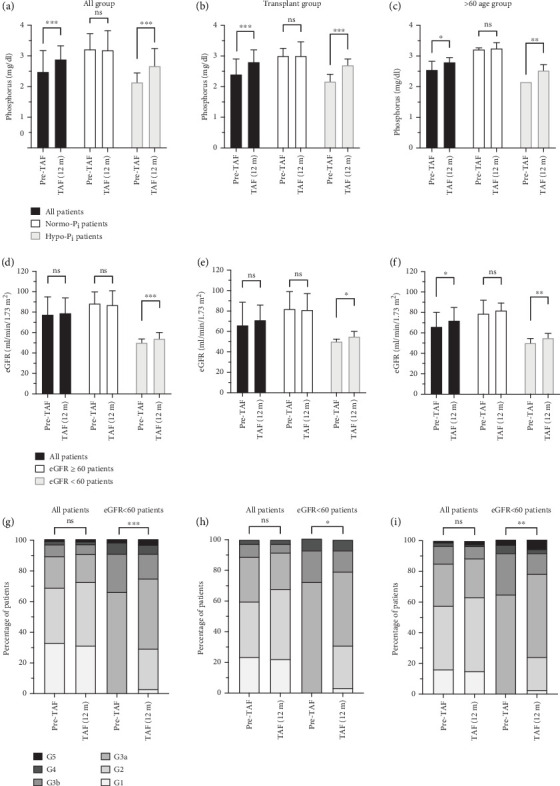
Phosphorus levels at Pre-TAF and TAF (12 m) in the total cohort (a), liver transplant recipients (b), and > 60 age patients (c). eGFR levels at Pre-TAF and TAF (12 m) in the total cohort (d), liver transplant recipients (e), and > 60 age patients (f). The alteration of eGFR stages from Pre-TAF to TAF (12 m) in all and eGFR < 60 patients among the total cohort (g), liver transplant recipient (h), and > 60 age patients (i). Pi and eGFR levels were shown as median (IQR), and stages of eGFR were shown as percentage of patients (%). Wilcoxon test. ns = not significant, ⁣^∗^*p* < 0.05,^∗∗^*p* < 0.01,^∗∗∗^*p* < 0.001 (eGFR, estimated glomerular filtration rate; G, grade; Hypo-Pi, Pi < 2.5 mg/dL; IQR, interquartile range; Normo-Pi, Pi ≥ 2.5 mg/dL; Pi, inorganic phosphorus).

In the > 60 age group, there was a statistically significant improvement from Pre-TAF to TAF (12 m) in the all patients group (*n* = 87) and the < 60 eGFR group (*n* = 37) (66 [51–80] vs. 72 [55–85] and 50 [41–54] vs. 55 [45–59] mL/min/1.73 m^2^; *p* = 0.033 and *p* = 0.006, respectively). In the ≥ 60 eGFR group (*n* = 50), eGFR levels were 79 (71–92) mL/min/1.73 m^2^ at Pre-TAF and 82 (73–89) mL/min/1.73 m^2^ at TAF (12 m); the difference was not statistically significant (*p* = 0.604). The proportion of eGFR stages in the all patients group was not statistically different from baseline values (*p* = 0.410). The alteration from Pre-TAF to TAF (12 m) in eGFR stages improved statistically significantly in the < 60 eGFR group (*p* = 0.002).  Figure 2 shows the changes in Pi levels, eGFR levels and stages of eGFR from Pre-TAF to TAF (12 m) among the total cohort, liver transplant recipients, and > 60 age patients.

### 3.3. Lipid Profile

**Figure 3:**
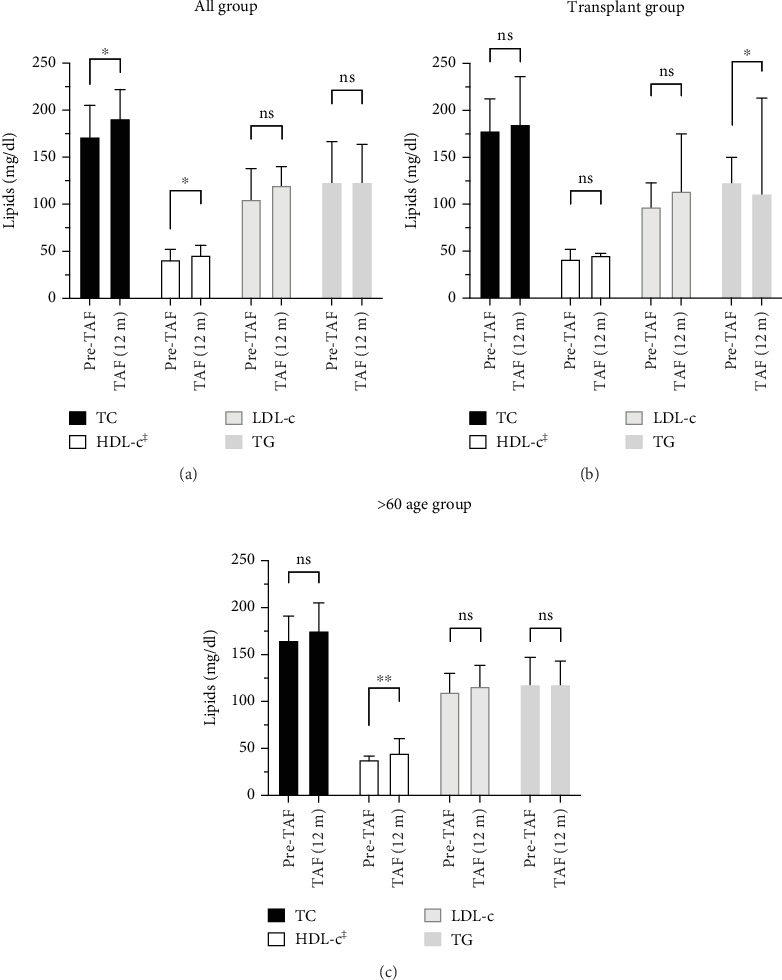
Lipid profile (TC, HDL-c, LDL-c, and TG) at Pre-TAF and TAF (12 m) in the total cohort (a), liver transplant recipients (b), and > 60 age patients (c). All data were shown as median (IQR). Wilcoxon test. ns = not significant, ⁣^∗^*p* < 0.05,^∗∗^*p* < 0.01. ^‡^all group (*n* = 41), transplant group (*n* = 10) and > 60 age group (*n* = 13) (HDL-c, high-density lipoprotein; IQR, interquartile range; LDL-c, low-density lipoprotein; TC, total cholesterol; TG, triglyceride).

As shown in Figure 3, lipid profiles of the total cohort, post-Tx, and > 60 age groups were evaluated by comparing Pre-TAF to TAF (12 m) levels of the TC, HDL-c, LDL-c, and TG. In the total cohort, TC levels (*n* = 52) were 171.5 (156–204.5) mg/dL at Pre-TAF and 191 (165.5–220) mg/dL at TAF (12 m); HDL-c levels (*n* = 41) were 42.98 ± 14.81 mg/dL at Pre-TAF and 46 (39–56) mg/dL at TAF (12 m); the differences were statistically significant (*p* = 0.015 and *p* = 0.023, respectively). The differences in LDL-c (*n* = 47) and TG (*n* = 56) levels from Pre-TAF to TAF (12 m) were not statistically significant in the total cohort, but a numerical increase in LDL-c levels was found (105 [84–137.5] mg/dL at Pre-TAF and 120 [97.5–139.5] mg/dL at TAF [12 m], 123 [86–165] mg/dL at Pre-TAF and 123 [95–163] mg/dL at TAF [12 m]; *p* = 0.147 and *p* = 0.163, respectively).

The change in the lipid profile of the post-Tx group from Pre-TAF to TAF (12 m) was statistically significant only for TG levels. TC levels (*n* = 14) were 178 (160–206) mg/dL at Pre-TAF and 185 (168–235) mg/dL at TAF (12 m) (*p* = 0.272). HDL-c levels (*n* = 10) changed from 41.5 (41–52) to 45.5 (39–47) mg/dL (*p* = 0.877). There was a slight numerical increase in LDL-c levels (*n* = 11) from Pre-TAF to TAF (12 m) (97 [89.5–120.5] vs. 114 [101–155.5] mg/dL; *p* = 0.241). TG levels (*n* = 15) decreased statistically significantly from Pre-TAF to TAF (12 m) (123 [83–148.5] vs. 111 [97.5–212] mg/dL; *p* = 0.015).

In the > 60 age group, the statistically significant difference from Pre-TAF to TAF (12 m) was seen in HDL-c levels, while no significant difference was observed in TC, LDL-c, and TG levels during the longitudinal follow-up. TC levels (*n* = 19) increased minimally from 165 (123–184.5) to 175 (159.5–204.5) mg/dL, but the difference did not reach statistical significance (*p* = 0.070). The change in HDL-c levels (*n* = 13) from Pre-TAF to TAF (12 m) was significant (38 [33–41] vs. 45 [38–56] mg/dL; *p* = 0.003). LDL-c levels (*n* = 17) were 110 (92–122) mg/dL at Pre-TAF and 116 (98–137) mg/dL at TAF (12 m); TG levels (*n* = 23) were 109 (77–141) mg/dL at Pre-TAF and 118 (88.5–138.5) mg/dL at TAF (12 m). The differences in these parameters were not statistically significant (*p* = 0.381 and *p* = 0.280, respectively).

### 3.4. Virological Response and ALT Normalization

Among the total cohort, the difference in HBV-DNA titers between Pre-TAF and TAF (12 m) improved statistically significantly in antiviral naïve patients (*p* = 0.011); there was also a statistical difference in treatment-experienced patients (*p* = 0.003). The ALT normalization rate increased in antiviral naïve patients but did not reach statistical significance (*p* = 0.275). In treatment-experienced patients, the rate of abnormal ALT levels was similar in Pre-TAF and TAF (12 m) (*p* = 0.789). Twenty-nine (90.6%) of 32 patients with elevated ALT at the initiation of TAF had normalized ALT after TAF treatment in treatment-experienced patients.

In post-Tx patients, the differences between Pre-TAF and TAF (12 m) in HBV-DNA titers and ALT normalization were not statistically significant (*p* = 0.317 and *p* = 0.827, respectively). There were no antiviral naïve patients among liver transplant recipients.

**Figure 4:**
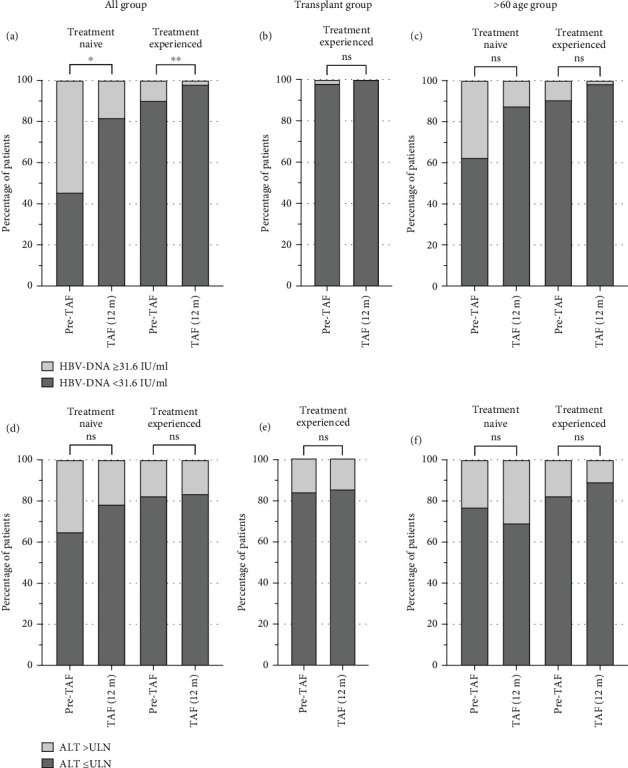
Alteration of HBV-DNA titer from Pre-TAF to TAF (12 m) in treatment naïve and experienced patients among the total cohort (a), liver transplant recipient (b), and > 60 age patients (c). The ALT normalization rate from Pre-TAF to TAF (12 m) in treatment naïve and experienced patients among the total cohort (d), liver transplant recipient (e), and > 60 age patients (f). All data were shown as percentage of patients (%). Wilcoxon test. ns = not significant, ⁣^∗^*p* < 0.05,^∗∗^*p* < 0.01 (ALT, alanine aminotransferase; ULN, upper limit of the normal range).

The change in HBV-DNA titers and ALT normalization in antiviral naïve patients among the > 60 age group was not statistically significant (*p* = 0.317 and *p* = 0.705, respectively). The difference in HBV-DNA titers from Pre-TAF to TAF (12 m) was not statistically significant in treatment-experienced patients among the > 60 age group despite numerical improvement was found, as naïve patients (*p* = 0.059). Likewise, the alteration in the rate of ALT normalization was not statistically significant (*p* = 0.275). The virological response and ALT normalization rate in patient subgroups are shown in Figure 4.

### 3.5. Other Outcomes

Cirrhosis did not develop in any noncirrhotic patients at baseline. None of the compensated cirrhotic patients developed any decompensation during the TAF treatment. Four patients had a worsening Child score, while another four patients had an improvement (*p* = 0.388). The MELD-Na score decreased in 13 patients, whereas increased in 8 patients from baseline to the last visit (*p* = 0.398). As shown in Table 2, Pi and transaminase levels statistically significant improved, while no significant change was detected in eGFR levels and stages among DC patients.

The alteration in the FIB-4 score stage of the patients in the total cohort was not statistically significant, with 20 patients improved and 16 patients worsened (*p* = 0.234). Changes in laboratory parameters not mentioned above in the total cohort are shown in Table 3. In patients who switched from TDF to TAF, statistically significant improvement was observed in virologic response, transaminase, eGFR, and Pi levels, whereas there was no change in eGFR stages and ALT normalization rate. Changes in laboratory parameters between Pre-TAF and TAF (12 m) in patients switched from TDF to TAF and from ETV to TAF, antiviral naïve, and HBsAg-positive patients are shown in Table S2, Table S3, Table S4, and Table S5, respectively.

The newly diagnosed HCC or deterioration of the previous disease was not seen during the TAF treatment. Loss of HBsAg, virological breakthrough, and acute hepatitis flare-up associated with HBV did not occur throughout the TAF treatment. No interruption or withdrawal of TAF treatment was required due to any serious adverse events related to TAF in the total cohort, even in patients with DC or liver transplant recipients.

## 4. Discussion

In this study, we evaluated the efficacy and safety of TAF treatment based on a real-world, single-center cohort. Our results revealed evidence particularly biochemical and virological responses of the TAF. In addition to treatment naïve patients in our cohort, the vast majority of patients were switched from TDF, and so these patients were primarily under the effect of TDF. The reasons for initiating or switching to TAF in this cohort also support this, given the impact of TDF on renal function and bone health. The isolated effect of the TAF on the patients with Hepatitis B was observed in antiviral naïve patients. This current study is remarkable in that it evaluates many patient subgroups in a single cohort, including frail patients such as those with DC, liver transplant recipients, and elderly individuals. Moreover, it stands out as a study that concurrently evaluated multiple aspects of TAF effect such as renal, metabolic, and antiviral efficacy in a single, quite large cohort. It provides evidence that favorable outcomes, in particular renal and antiviral efficacy, can be demonstrated even in vulnerable patient populations.

The development of hypophosphatemia in patients using TDF has been shown in the literature [14]. In prior reports on switching from TDF to TAF, Pi levels did not change significantly, but one study also found remaining renal phosphate wasting and worsening of Pi levels after the TAF treatment [15–17]. No significant difference was found between ETV and TAF regarding Pi levels [18–21]. Apart from these studies, our results showed that significant improvements in Pi levels occurred in distinct groups of patients. Besides this favorable impact, it did not exceed the threshold value (2.5 mg/dL) in elderly individuals. In addition, we can speculate that the improvement in alkaline phosphatase levels may be due to the improvement in osteomalacia with a reduction in renal phosphorus loss. Further research is warranted to confirm this hypothesis. TAF could be a potential therapeutic agent, especially in hypophosphatemic patients or to prevent Pi depletion.

According to the results of the current study, TAF gave favorable outcomes in terms of renal function in patients with low eGFR, particularly in elderly patients. In most cases, TAF was found to be more beneficial than TDF regarding renal dysfunction, whereas there was no such obvious data for ETV [7, 8, 15–23]. The renal outcomes in our study were consistent with the majority of the literature. Consequently, we consider that TAF can be safely used in patients with chronic kidney disease.

The effect of TAF on metabolic parameters such as lipid profile is controversial in the literature. In general, TDF can improve the lipid profile, but recent studies have provided some evidence that TAF has a negative effect on the lipid profile [10, 15, 22, 24]. Suzuki et al. reported worsening lipid levels based on the NCEP-ATP III criteria [10]. Additionally, there are also studies showing that the levels of TC, LDL-c, and HDL-c did not change significantly [23, 25]. Our findings that seem unfavorable in some patient groups contributed to the discrepancies in the literature. Additional analyses are required to assess the effect of changes in lipid profile on cardiovascular risk. Patients on TAF should be closely monitored for lipid profile to minimize cardiovascular risk in light of our data.

Previous studies have shown that TDF and TAF are similar in terms of virological response [7, 8, 15, 16, 22, 24]. Also, some researchers asserted that TAF was superior to TDF considering viral DNA suppression [26]. It was reported that TAF is a more potent suppressor for HBV-DNA compared with ETV, whereas similar efficacy was found in a recent study [19, 22, 27]. Treatment-naïve patients demonstrated a strong and sustained antiviral effect of TAF; even in treatment-experienced patients, significantly stronger viral suppression was achieved in our cohort. ALT normalization was higher with TAF than with ETV and TDF, but there are also studies where no significant change was observed [15, 16, 18, 19, 22, 24, 26, 27]. In our cohort, the ALT normalization rate did not change significantly, but ALT and AST levels decreased after TAF treatment. Therefore, TAF was found to be highly effective in suppressing HBV-DNA and may be useful in normalizing transaminase elevations.

Data on the effect of TAF in patients with DC are lacking in the literature. Patients with DC are more vulnerable in terms of mortality and morbidity; for instance, they are prone to acute and chronic kidney injury [28–30]. In this study, we showed that TAF is beneficial or neutral in terms of either various biochemical or virological outcomes in this patient group. Our results indicate that TAF can be used safely and effectively in patients with DC.

This study has several limitations, including its retrospective nature, single-center design, and some missing data. Changes in bone mineral density, proteinuria, and weight after TAF treatment could not be evaluated due to insufficient data. An independant evaluation of the effects of TAF in patients with DC, elderly individuals, and liver transplant recipients is also a prominent aspect of our study. Further investigations are required to reveal the efficacy and safety of TAF treatment, and our findings should be validated in larger cohorts.

In conclusion, TAF was safe, well tolerated, and effective in this real-world cohort consisting of patients with Hepatitis B. According to our results, TAF may be preferable compared to TDF in terms of virological and biochemical parameters, but there are insufficient data for ETV. TAF may be a favored option especially in elderly patients, patients with DC, renal dysfunction, and electrolyte imbalance.

## Figures and Tables

**Table 1:** Patients' baseline clinical and treatment characteristics, at onset of TAF.

Age, years, med (IQR) (*n* = 225)	57 (48–65)		
> 60 years, *n* (%) (*n* = 225)	99 (44)		
Follow-up period, months, med (IQR) (*n* = 225)	22 (12–30)		
Sex, male, *n* (%) (*n* = 225)	158 (70.2)		
Cirrhosis, *n* (%) (*n* = 194)	43 (22.2)		
Decompensated, *n* (%) (*n* = 43)	25 (58.1)		
MELD-Na, med (IQR) (*n* = 36)	11 (9–15.5)		
Child–Pugh class, *n* (%) (*n* = 43)	A	B	C
	18 (42)	20 (46)	5 (12)
FIB-4, *n* (%) (*N* = 221)	< 1.45	1.45–3.25	> 3.25
	110 (50)	75 (34)	36 (16)
Posttransplant, *n* (%) (*n* = 225)	72 (32)		
HBsAg positivity, *n* (%) (*n* = 225)	149 (66.2)		
HBeAg positivity, *n* (%) (*n* = 122)	10 (8.2)		
HBV-DNA ≥ 31.6 IU/mL, *n* (%) (*n* = 174)	27 (15.5)		
ALT > ULN, *n* (%) (*n* = 224)	46 (20.5)		
Total bilirubin, med (IQR), mg/dL (*n* = 198)	0.6 (0.37–0.97)		
Platelet, ×10^3^/*μ*L, med (IQR) (*n* = 222)	138 (180–239)		
<90 × 10^3^/*μ*L, *n* (%)	25 (11.3)		
Albumin, g/dL, med (IQR) (*n* = 212)	4.6 (4.27–4.8)		
< 3.5 g/dL, *n* (%)	17 (8)		
Prophylaxis to prevent reactivation, *n* (%) (*n* = 225)	41 (18.2)		
HBsAg positivity, *n* (%)	14 (34.1)		
Anti-HBc positivity in HBsAg negatives, *n* (%)	27 (65.9)		
Antiviral naïve, *n* (%) (*n* = 225)	39 (17.3)		
Pre-TAF antiviral duration, months, med (IQR) (*n* = 162)	127 (81–177)		
Switch to TAF, *n* (%) (*n* = 186)			
TDF	164 (88.2)		
ETV	18 (9.7)		
Others	4 (2)		
Causes of start/switch to TAF, *n* (%) (*n* = 225)			
Hypophosphatemia	110 (48.9)		
eGFR < 60 mL/min/1.73 m^2^	62 (27.6)		
Osteoporosis	22 (9.8)		
Proteinuria	5 (2.2)		
Others	17 (7.6)		

Abbreviations: ALT, alanine aminotransferase; eGFR, estimated glomerular filtration rate; ETV, entecavir; IQR, interquartile range; med, median; *n*, number of patients meet the criteria; *N*, number of evaluable patients; TAF, tenofovir alafenamide; TDF, tenofovir disoproxil fumarate; ULN, upper limit of the normal range.

**Table 2:** Changes in laboratory parameters after the TAF in patients with DC.

	**Pre-TAF**	**TAF (12** m**)**	**p** **-value** ^**a**^
HBV-DNA ≥ 31.6 IU/mL, *n* (%) (*n* = 16)	3 (18.8)	2 (12.5)	0.773
BUN, mg/dL, med (IQR) (*n* = 19)	18 (14–26.55)	21 (11–23.7)	0.760
Crea, mg/dL, med (IQR) (*n* = 24)	0.88 (0.75–1.25)	0.85 (0.74–1.32)	0.808
eGFR, mL/min/1.73 m^2^, med (IQR) (*n* = 24)	78.5 (57.5–97.5)	81 (58.5–95)	0.819
eGFR stages, *n* (%) (*n* = 24)			0.417
G1	10 (41.7)	8 (33.3)	
G2	8 (33.3)	9 (37.5)	
G3a	6 (25)	6 (25)	
G3b		1 (4.2)	
G4			
G5			
Pi, mg/dL, med (IQR) (*n* = 22)	2.6 (2.1–3.2)	2.8 (2.45–3.5)	**0.019**
Ca, mg/dL, mean ± SD (*n* = 23)	8.68 ± 0.59	8.91 ± 0.59	0.321
Na, mmol/L, med (IQR) (*n* = 23)	138 (137–139)	138 (136–140)	0.783
K, mmol/L, med (IQR) (*N* = 23)	4.14 (3.67–4.41)	4.5 (4–4.67)	0.080
Mg, mmol/L, med (IQR) (*N* = 18)	0.8 (0.78–0.84)	0.8 (0.72–0.92)	0.131
AST, U/L, med (IQR) (*n* = 24)	37 (25.5–70.5)	30.5 (23.7–43.8)	**0.013**
ALT, U/L, med (IQR) (*n* = 24)	24.5 (19–42.5)	23.8 (15.25–31.5)	**0.038**
ALT > ULN, *n* (%) (*n* = 24)	9 (37.5)	3 (12.5)	0.058
ALP, U/L, med (IQR) (*n* = 22)	101 (79–131)	109 (79–140)	0.770
GGT, U/L, med (IQR) (*n* = 24)	35 (21–71)	31.5 (17.5–54)	0.558
T.Bil, mg/dL, med (IQR) (*n* = 24)	1.87 (0.9–3.69)	1.11 (0.71–2.18)	0.114
Alb, g/dL, med (IQR) (*n* = 24)	3.7 (2.78–4.29)	3.8 (3.27–4.17)	0.898
INR, med (IQR) (*n* = 23)	1.36 (1.3–1.43)	1.26 (1.12–1.42)	0.086
WBC, /*μ*L, med (IQR) (*n* = 24)	4800 (3725–5150)	4150 (3400–6100)	0.797
Neu, /*μ*L, med (IQR) (*n* = 21)	2400 (1900–3300)	2400 (1800–3400)	0.465
Lymp, /*μ*L, med (IQR) (*n* = 21)	800 (530–1200)	800 (700–1400)	0.628
Plt, ×10^3^/*μ*L, med (IQR) (*n* = 24)	68 (54–114.5)	84.5 (56–122.5)	0.241

*Note:* Changes in laboratory parameters from Pre-TAF to TAF (12 m) in patients with decompensated cirrhosis.

Abbreviations: Alb, albumine; ALP, alkaline phospatase; ALT, alanine aminotransferase; AST, aspartate aminotransferase; BUN, blood urea nitrogen; Ca, calcium; Crea, creatinine; DC, decompensated cirrhosis; eGFR, estimated glomerular filtration rate; GGT, gamma glutamyl transferase; INR, international normalized ratio; IQR, interquartile range; K, potassium; Lymp, lymphocyte; med, median; Mg, magnesium; *n*, number of patients meet the criteria; *N*, number of evaluable patients; Na, sodium; Neu, neutrophil; Pi, inorganic phosphorus; Plt, platelet; SD, standard deviation; T. Bil, total bilirubin; ULN, upper limit of normal; WBC, white blood cell.

^a^Wilcoxon test.

**Table 3:** Changes in laboratory parameters after the TAF in the total cohort.

	**Pre-TAF**	**TAF (12** m**)**	**p** **-value** ^**a**^
BUN, mg/dL, med (IQR) (*n* = 169)	16.4 (13.0–22.15)	16.3 (13.05–22.0)	0.804
Crea, mg/dL, med (IQR) (*n* = 220)	1.01 (0.81–1.30)	0.99 (0.80–1.25)	**0.009**
Ca, mg/dL, med (IQR) (*n* = 206)	9.40 (9.1–9.7)	9.4 (9.1–9.7)	0.420
Na, mmol/L, med (IQR) (*n* = 203)	140 (138–142)	140 (138–142)	0.137
K, mmol/L, med (IQR) (*n* = 202)	4.30 (4.0–4.67)	4.50 (4.17–4.70)	**0.003**
Mg, mmol/L, med (IQR) (*n* = 162)	0.82 (0.77–0.90)	0.83 (0.79–0.91)	**0.039**
ALT, U/L, med (IQR) (*n* = 219)	21 (15–31)	18 (14–26)	**< 0.001**
AST, U/L, med (IQR) (*n* = 219)	22 (17–29)	20 (16–25)	**< 0.001**
ALP, U/L, med (IQR) (*n* = 207)	87 (71–114)	84 (67–104)	**0.021**
GGT, U/L, med (IQR) (*n* = 208)	21 (14–33)	20 (13–31)	0.891
T. Bil, mg/dL, med (IQR) (*n* = 184)	0.6 (0.38–0.98)	0.57 (0.4–0.91)	0.062
Alb, g/dL, med (IQR) (*n* = 205)	4.6 (4.26–4.8)	4.5 (4.20–4.64)	**< 0.001**
INR, med (IQR) (*n* = 158)	1.0 (0.94–1.15)	1.02 (0.95–1.10)	0.699
WBC, /*μ*L, med (IQR) (*n* = 215)	6550 (5020–8200)	6500 (5000–8400)	0.585
Neu, /*μ*L, med (IQR) (*n* = 200)	3950 (2900–5075)	3950 (2725–5075)	0.952
Lymp, /*μ*L, med (IQR) (*n* = 195)	1600 (1200–2100)	1700 (1300–2200)	**0.029**
Plt, ×10^3^/*μ*L, med (IQR) (*n* = 215)	180 (135–237)	204 (143–252)	**< 0.001**

*Note:* Changes in laboratory parameters from Pre-TAF to TAF (12 m) in the total cohort.

Abbreviations: Alb, albumine; ALP, alkaline phospatase; ALT, alanine aminotransferase; AST, aspartate aminotransferase; BUN, blood urea nitrogen; Ca, calcium; Crea, creatinine; GGT, gamma glutamyl transferase; INR, international normalized ratio; IQR, interquartile range; K, potassium; Lymp, lymphocyte; Mg, magnesium; *N*, number of evaluable patients; Na, sodium; Neu, neutrophil; Plt, platelet; SD, standard deviation; T. Bil, total bilirubin; WBC, white blood cell.

^a^Wilcoxon test.

## Data Availability

The data that support the findings of this study are available from the corresponding author upon reasonable request.

## Supporting Information

Supporting Information Additional supporting information can be found online in the Supporting Information section. Table S1 presents the causes of switch from other NAs to TAF, Table S2 presents the changes in laboratory parameters in switch from TDF to TAF patients, Table S3 presents the changes in laboratory parameters in switch from ETV to TAF patients, Table S4 presents the changes in laboratory parameters in antiviral naïve patients, and Table S5 presents the changes in laboratory parameters in HBsAg-positive patients.
