# Supplementary material for: Virological and Biochemical Effects of Tenofovir Alafenamide in Different Patient Groups With Chronic Hepatitis B Virus Infection in Real-World Cohort
Source: Int J Hepatol. 2025 Apr 15;2025:9632839. doi: 10.1155/ijh/9632839 (PMC12014250; doi:10.1155/ijh/9632839)
Supplement: Supporting Information — Additional supporting information can be found online in the Supporting Information section. Table S1 presents the causes of switch from other NAs to TAF, Table S2 presents the changes in laboratory parameters in switch from TDF to TAF patients, Table S3 presents the changes in laboratory parameters in switch from ETV to TAF patients, Table S4 presents the changes in laboratory parameters in antiviral naïve patients, and Table S5 presents the changes in laboratory parameters in HBsAg-positive patients. [file 9632839.f1.pdf]

Supplementary Materials

Supplementary Table S1. Causes of switch from other NAs to TAF

	NAs to TAF (N=186)	TDF to TAF (N=164)	ETV to TAF (N=18)	Others to TAF (N=4)	p-value‡
**Hypo-P_i, n (%)**	100 (53.8)	93 (56.7)	5 (27.8)	2 (50)	**0.039**
**eGFR <60, n (%)**	52 (28)	44 (26.8)	5 (27.8)	3 (75)	1.000
**Osteoporosis, n (%)**	21 (11.3)	18 (11)	1 (5.6)	2 (50)	0.698
**Proteinuria, n (%)**	5 (2.7)	4 (2.4)	1 (5.6)	0 (0)	0.410
**Others, n (%)**	16 (8.6)	7 (4.3)	8 (44.4)	1 (25)	**<0.001**
**≥2 causes, n (%)**	24 (12.9)	19 (11.6)	2 (11.1)	3 (75)	1.000

Anti-viral switching causes from NAs (TDF, ETV and others) to TAF, from TDF to TAF, from ETV to TAF and from others to TAF patients. ‡TDF to TAF vs ETV to TAF, Chi-square or Fischer's exact test, as appropriate. eGFR was measured in ml/min/1.73m^2. Abbreviations: eGFR, estimated glomerular filtration rate; ETV, entecavir; Hypo-P_i, hypophosphatemia; N, number of evaluable patients; NAs, nucleotide analogs; TAF, tenofovir alafenamide; TDF, tenofovir disoproxil fumarate.

Supplementary Table S2. Changes in laboratory parameters in switch from TDF to TAF patients

	Pre-TAF	TAF (12 m)	p-value[¶]
**HBV DNA ≥31.6 IU/ml, n (%) (n=134)**	9 (6.7)	2 (1.5)	**0.035**
**BUN, mg/dL, med (IQR) (n=117)**	16 (13-21.2)	16 (13.1-21.3)	0.794
**Crea, mg/dL, med (IQR) (n=160)**	1.07 (0.88-1.32)	1 (0.82-1.25)	**<0.001**
**eGFR, ml/min/1.73m^2, med (IQR) (n=160)**	73 (54.5-94)	77.5 (58-90)	**0.047**
**eGFR stages, n (%) (n=160)**			0.326
**G1**	48 (30)	42 (26.3)	
**G2**	57 (35.6)	74 (46.3)	
**G3a**	38 (23.8)	30 (18.8)	
**G3b**	12 (7.5)	10 (6.3)	
**G4**	5 (3.1)	3 (1.9)	
**G5**		1 (0.6)	
**P_i, mg/dL, med (IQR) (n=149)**	2.4 (2.1-2.9)	2.74 (2.47-3.2)	**<0.001**
**Ca, mg/dL, med (IQR) (n=154)**	9.4 (9.1-9.7)	9.38 (9.13-9.65)	0.717
**Na, mmol/L, med (IQR) (n=149)**	140 (138-142)	140 (138-142)	0.067
**K, mmol/L, med (IQR) (n=149)**	4.3 (4-4.66)	4.49 (4.18-4.67)	**0.004**
**Mg, mmol/L, med (IQR) (n=117)**	0.82 (0.77-0.9)	0.82 (0.79-0.9)	0.131
**AST, U/L, med (IQR) (n=160)**	23 (19-28.5)	21 (16-25.4)	**<0.001**
**ALT, U/L, med (IQR) (n=160)**	21 (15-29.9)	18.5 (14.2-25)	**0.001**
**ALT >ULN, n (%) (n=160)**	28 (17.5)	28 (17.5)	1.000
**ALP, U/L, med (IQR) (n=154)**	87.5 (72-117)	84 (67-104)	**0.003**
**GGT, U/L, med (IQR) (n=158)**	20.5 (14-32)	20 (14-31)	0.227
**T.Bil, mg/dL, med (IQR) (n=142)**	0.6 (0.38-0.97)	0.57 (0.4-0.92)	0.123
**Alb, g/dL, med (IQR) (n=151)**	4.6 (4.32-4.8)	4.5 (4.3-4.64)	**<0.001**
**INR, med (IQR) (n=118)**	1 (0.94-1.13)	1.02 (0.95-1.1)	0.943
**WBC, /μL, med (IQR) (n=158)**	6400 (5000-7700)	6400 (5100-8200)	**0.048**
**Neu, /μL, med (IQR) (n=146)**	3800 (2900-5000)	4000 (2800-5000)	0.285
**Lymp, /μL, med (IQR) (n=143)**	1500 (1200-2000)	1700 (1200-2200)	**0.014**
**Plt, x10^3/μL, med (IQR) (n=158)**	168.5 (127-230)	196 (141-235)	**<0.001**

Changes in laboratory parameters from Pre-TAF to TAF (12 m) in patients that switch from TDF to TAF. [¶]Wilcoxon test. Abbreviations: ALT, alanine aminotransferase; ALP, alkaline phospatase; Alb, albumine; AST, aspartate aminotransferase; BUN, blood urea nitrogen; Ca, calcium; Crea, creatinine; DC, decompensated cirrhosis; eGFR, estimated glomerular filtration rate; GGT, gamma glutamyl transferase; IQR, interquartile range; INR, international normalized ratio; K, potassium; Lymp, lymphocyte**;** med, median; Mg, magnesium; Na, sodium; Neu, neutrophil; n, number of patients meet the criteria; N, number of evaluable patients; P_i, inorganic phosphorus; Plt, platelet; SD, standard deviation; TAF, tenofovir alafenamide; TDF, tenofovir disoproxil fumarate; T. Bil, total bilirubin; ULN, upper limit of normal; WBC, white blood cell.

Supplementary Table S3. Changes in laboratory parameters in switch from ETV to TAF patients

	Pre-TAF	TAF (12 m)	p-value¶
**HBV DNA ≥31.6 IU/ml, n (%) (n=15)**	6 (40)	1 (6.7)	**0.025**
**BUN, mg/dL, med (IQR) (n=16)**	17.1 (14.5-29)	15.2 (13.5-26.1)	0.615
**Crea, mg/dL, med (IQR) (n=18)**	1.01 (0.83-1.3)	1.08 (0.8-1.32)	0.887
**eGFR, ml/min/1.73m^2, med (IQR) (n=18)**	73.5 (58-92)	56 (72-92)	0.955
**eGFR stages, n (%) (n=18)**			0.564
**G1**	5 (27.8)	5 (27.8)	
**G2**	8 (44.4)	7 (38.9)	
**G3a**	3 (16.7)	4 (22.2)	
**G3b**	1 (5.6)	1 (5.6)	
**G4**			
**G5**	1 (5.6)	1 (5.6)	
**P_i, mg/dL, med (IQR) (n=17)**	3.3 (2.45-3.7)	3.05 (2.8-3.4)	0.586
**Ca, mg/dL, med (IQR) (n=16)**	9.6 (9.38-9.74)	9.34 (9.15-9.64)	0.234
**Na, mmol/L, med (IQR) (n=17)**	141 (139-142)	140 (139-141)	0.433
**K, mmol/L, med (IQR) (n=16)**	4.25 (3.96-4.66)	4.32 (3.99-4.68)	0.698
**Mg, mmol/L, med (IQR) (n=14)**	0.84 (0.8-0.9)	0.89 (0.8-0.94)	0.683
**AST, U/L, med (IQR) (n=18)**	17.5 (15-23)	18.7 (14-23.6)	0.983
**ALT, U/L, med (IQR) (n=18)**	18.5 (11-24)	21.25 (11-31)	0.959
**ALT >ULN, n (%) (n=18)**	4 (22.2)	2 (11.1)	0.317
**ALP, U/L, med (IQR) (n=16)**	87 (72-108.5)	85.5 (63-101.5)	0.836
**GGT, U/L, med (IQR) (n=16)**	21.5 (16.5-32)	23.5 (14-32.5)	0.754
**T.Bil, mg/dL, med (IQR) (n=15)**	0.7 (0.48-0.83)	0.56 (0.44-0.63)	0.594
**Alb, g/dL, med (IQR) (n=17)**	4.6 (4-4.8)	4.6 (3.9-4.86)	0.740
**INR, med (IQR) (n=13)**	0.94 (0.9-1.04)	0.95 (0.9-1.02)	0.224
**WBC, /μL, med (IQR) (n=17)**	6700 (5700-8100)	6700 (5500-7900)	0.554
**Neu, /μL, med (IQR) (n=16)**	4100 (3250-4700)	3600 (2950-4650)	0.570
**Lymp, /μL, med (IQR) (n=14)**	2000 (1200-2300)	1900 (1400-2100)	0.361
**Plt, x10^3/μL, med (IQR) (n=17)**	180 (161-254)	220 (171-255)	0.061

Changes in laboratory parameters from Pre-TAF to TAF (12 m) in patients that switch from ETV to TAF. ¶Wilcoxon test. Abbreviations: ALT, alanine aminotransferase; ALP, alkaline phospatase; Alb, albumine; AST, aspartate aminotransferase; BUN, blood urea nitrogen; Ca, calcium; Crea, creatinine; DC, decompensated cirrhosis; eGFR, estimated glomerular filtration rate; ETV, entecavir; GGT, gamma glutamyl transferase; IQR, interquartile range; INR, international normalized ratio; K, potassium; Lymp, lymphocyte**;** med, median; Mg, magnesium; Na, sodium; Neu, neutrophil; n, number of patients meet the criteria; N, number of evaluable patients; P_i, inorganic phosphorus; Plt, platelet; SD, standard deviation; TAF, tenofovir alafenamide; T. Bil, total bilirubin; ULN, upper limit of normal; WBC, white blood cell.

Supplementary Table S4. Changes in laboratory parameters in anti-viral naive patients

	Pre-TAF	TAF (12 m)	p-value¶
**HBV DNA ≥31.6 IU/ml, n (%) (n=22)**	12 (54.5)	4 (18.2)	**0.011**
**BUN, mg/dL, med (IQR) (n=32)**	16.7 (12.8-21.9)	16.6 (12.8-22.8)	0.899
**Crea, mg/dL, med (IQR) (n=38)**	0.81 (0.7-1.03)	0.84 (0.68-1.09)	0.523
**eGFR, ml/min/1.73m^2, med (IQR) (n=38)**	90.5 (79-100)	93.5 (76-104)	0.717
**eGFR stages, n (%) (n=38)**			0.635
**G1**	20 (52.6)	22 (57.9)	
**G2**	13 (34.2)	9 (23.7)	
**G3a**	3 (7.9)	4 (10.5)	
**G3b**	2 (5.3)	2 (5.3)	
**G4**		1 (2.6)	
**G5**			
**P_i, mg/dL, mean ± SD (n=26)**	3.24 ± 0.72	3.48 ± 0.59	0.137
**Ca, mg/dL, med (IQR) (n=32)**	9.39 (9-9.66)	9.50 (8.94-9.8)	0.911
**Na, mmol/L, med (IQR) (n=33)**	140 (138-142)	141 (139-142)	0.495
**K, mmol/L, med (IQR) (n=33)**	4.34 (4-4.6)	4.58 (4.21-4.87)	0.169
**Mg, mmol/L, med (IQR) (n=28)**	0.85 (0.74-0.9)	0.85 (0.8-0.91)	0.121
**AST, U/L, med (IQR) (n=37)**	21.5 (14.6-39.9)	18.8 (15-23.4)	0.080
**ALT, U/L, med (IQR) (n=37)**	23 (17-48)	16.5 (13.8-26.7)	**0.006**
**ALT >ULN, n (%) (n=37)**	13 (35.1)	8 (21.6)	0.275
**ALP, U/L, med (IQR) (n=33)**	71 (62-101)	78 (70-109)	0.519
**GGT, U/L, med (IQR) (n=30)**	26.5 (13-52)	22 (13-31)	**0.029**
**T.Bil, mg/dL, med (IQR) (n=23)**	0.47 (0.37-1.14)	0.6 (0.33-0.8)	0.223
**Alb, g/dL, med (IQR) (n=33)**	4.39 (3.8-4.59)	4.3 (4.05-4.56)	0.865
**INR, med (IQR) (n=23)**	1.06 (0.98-1.25)	1.05 (1.02-1.11)	0.445
**WBC, /μL, med (IQR) (n=36)**	7600 (5550-9900)	7350 (4400-8735)	0.198
**Neu, /μL, med (IQR) (n=34)**	4150 (3100-5900)	3900 (2400-5300)	0.386
**Lymp, /μL, med (IQR) (n=34)**	1900 (1400-2400)	2012 ± 686¥	0.411
**Plt, x10^3/μL, med (IQR) (n=36)**	222 (183-259)	252(191-294)	0.203

Changes in laboratory parameters from Pre-TAF to TAF (12 m) in anti-viral naive patients. ¶Wilcoxon test. ¥Data were showed mean ± SD because of normal distribution. Abbreviations: ALT, alanine aminotransferase; ALP, alkaline phospatase; Alb, albumine; AST, aspartate aminotransferase; BUN, blood urea nitrogen; Ca, calcium; Crea, creatinine; DC, decompensated cirrhosis; eGFR, estimated glomerular filtration rate; GGT, gamma glutamyl transferase; IQR, interquartile range; INR, international normalized ratio; K, potassium; Lymp, lymphocyte; med, median; Mg, magnesium; Na, sodium; Neu, neutrophil; n, number of patients meet the criteria; N, number of evaluable patients; P_i, inorganic phosphorus; Plt, platelet; SD, standard deviation; TAF, tenofovir alafenamide; T. Bil, total bilirubin; ULN, upper limit of normal; WBC, white blood cell.

Supplementary Table S5. Changes in laboratory parameters in HBsAg positive patients

	Pre-TAF	TAF (12 m)	p-value¶
**HBV DNA ≥31.6 IU/ml, n (%) (n=122)**	27 (22.1)	7 (5.7)	**<0.001**
**BUN, mg/dL, med (IQR) (n=110)**	16.1 (13-22)	16.2 (13-22)	0.459
**Crea, mg/dL, med (IQR) (n=144)**	1 (0.8-1.3)	0.96 (0.8-1.3)	0.137
**eGFR, ml/min/1.73m^2, med (IQR) (n=144)**	77.5 (56.6-96.5)	81 (58-94)	0.328
**eGFR stages, n (%) (n=144)**			0.579
**G1**	53 (36.8)	47 (32.6)	
**G2**	49 (34)	55 (38.2)	
**G3a**	26 (18.1)	27 (18.8)	
**G3b**	11 (7.6)	11 (7.6)	
**G4**	4 (2.8)	2 (1.4)	
**G5**	1 (0.7)	2 (1.4)	
**P_i, mg/dL, med (IQR) (n=128)**	2.44 (2.16-3.2)	2.87 ± 0.67¥	**<0.001**
**Ca, mg/dL, med (IQR) (n=135)**	9.38 (9.1-9.67)	9.39 (9.1-9.63)	0.527
**Na, mmol/L, med (IQR) (n=130)**	140 (138-142)	140 (138-142)	0.113
**K, mmol/L, med (IQR) (n=130)**	4.26 (4-4.67)	4.45 (4.1-4.7)	**<0.001**
**Mg, mmol/L, med (IQR) (n=103)**	0.84 (0.78-0.9)	0.85 (0.8-0.92)	**0.038**
**AST, U/L, med (IQR) (n=143)**	23 (19-29.5)	21 (17-26.3)	**<0.001**
**ALT, U/L, med (IQR) (n=143)**	22 (15-32.5)	19 (14.1-26)	**<0.001**
**ALT >ULN, n (%) (n=143)**	35 (24.5)	23 (16.1)	0.096
**ALP, U/L, med (IQR) (n=135)**	85 (70.5-105)	84 (65.5-104)	0.087
**GGT, U/L, med (IQR) (n=139)**	21 (14-32)	20 (13-28.5)	0.468
**T.Bil, mg/dL, med (IQR) (n=122)**	0.6 (0.4-0.98)	0.6 (0.42-0.85)	0.086
**Alb, g/dL, med (IQR) (n=134)**	4.55 (4.26-4.8)	4.5 (4.2-4.62)	**0.001**
**INR, med (IQR) (n=102)**	1.05 (0.98-1.24)	1.03 (0.97-1.15)	0.099
**WBC, /μL, med (IQR) (n=140)**	6561 ± 2183¥	6400 (4800-8000)	0.967
**Neu, /μL, med (IQR) (n=127)**	4000 (2900-4900)	3700 (2650-4900)	0.595
**Lymp, /μL, med (IQR) (n=125)**	1700 (1200-2100)	1700 (1300-2200)	0.640
**Plt, x10^3/μL, med (IQR) (n=140)**	186 (132-237)	204 (130-250)	**0.001**

Changes in laboratory parameters from Pre-TAF to TAF (12 m) in HBsAg positive patients. ¶Wilcoxon test. ¥Data were showed mean ± SD because of normal distribution. Abbreviations: ALT, alanine aminotransferase; ALP, alkaline phospatase; Alb, albumine; AST, aspartate aminotransferase; BUN, blood urea nitrogen; Ca, calcium; Crea, creatinine; DC, decompensated cirrhosis; eGFR, estimated glomerular filtration rate; GGT, gamma glutamyl transferase; IQR, interquartile range; INR, international normalized ratio; K, potassium; Lymp, lymphocyte; med, median; Mg, magnesium; Na, sodium; Neu, neutrophil; n, number of patients meet the criteria; N, number of evaluable patients; P_i, inorganic phosphorus; Plt, platelet; SD, standard deviation; TAF, tenofovir alafenamide; T. Bil, total bilirubin; ULN, upper limit of normal; WBC, white blood cell.
